# Multioutput Machine Learning Model for Predicting Postoperative Outcomes After Liposuction: Algorithm Development and Validation Study in a Multicenter Cohort

**DOI:** 10.2196/82145

**Published:** 2026-09-23

**Authors:** Chaewoo Lee, Seoyoung Park, Jiyoung Hwang, Selin Woo, Youn Chan Park, Jae Won Seo, Sun Ho Lee, Jae Hyun Ahn, Dong Keon Yon, Sang Youl Rhee

**Affiliations:** 1 Department of Computer Science and Engineering, Seoul Metropolitan University College of Engineering, Seoul, Republic of Korea; 2 Center for Digital Health, Medical Science Research Institute, Kyung Hee University Medical Center, Kyung Hee University College of Medicine, 23 Kyungheedae-ro, Dongdaemun-gu, Seoul, 02447, Republic of Korea, 82 2-961-0680, 82 504-478-0201; 3 Department of Precision Medicine, Kyung Hee University College of Medicine, Seoul, Republic of Korea; 4 Department of Medicine, Kyung Hee University College of Medicine, Seoul, Republic of Korea; 5 Busan 365mc Hospital, Busan, Republic of Korea; 6 Daegu 365mc Hospital, Daegu, Republic of Korea; 7 Daejeon 365mc Hospital, Daejeon, Republic of Korea; 8 Incheon 365mc Hospital, Incheon, Republic of Korea; 9 Department of Pediatrics, Kyung Hee University Medical Center, Kyung Hee University College of Medicine, Seoul, Republic of Korea; 10 Department of Endocrinology and Metabolism, Kyung Hee University College of Medicine, Seoul, Republic of Korea

**Keywords:** artificial intelligence, AI, liposuction, machine learning, multioutput, obesity, postoperative

## Abstract

**Background:**

Liposuction is widely performed to remove localized fat deposits and improve body contour, yet individualized prediction of postoperative outcomes remains challenging. Existing machine learning (ML) studies have largely focused on single-outcome prediction, with limited attention to the interdependence between postoperative body weight and circumferential size.

**Objective:**

This study aimed to develop and validate a chained multioutput ML framework to jointly predict postoperative body weight and circumferential size after liposuction using a large multicenter cohort from the 365mc network.

**Methods:**

We analyzed a multicenter cohort of 7804 individuals who underwent liposuction in 2024 at 20 obesity specialty clinics in the 365mc network across South Korea. Using 15 predictors, we compared 8 individual ML models, an automated ML approach, 2 ensemble approaches, and chained multioutput regression models for predicting postoperative body weight and circumferential size. Models were developed using 5-fold cross-validation and evaluated on an independent test set. Performance was assessed using the coefficient of determination (*R*^2^), root mean square error (RMSE), mean absolute error (MAE), and mean absolute percentage error (MAPE), and feature importance was evaluated using Shapley additive explanation (SHAP) values. The selected model was integrated into a web-based clinical decision support system (CDSS).

**Results:**

A total of 7804 individuals who underwent liposuction were included; of these, 7612 (97.54%) were female. The chained extra trees regressor model with a weight-to-size prediction order achieved an *R*^2^ of 0.98, an RMSE of 2.36, an MAE of 1.24, and a MAPE of 2.19. The SHAP analysis identified preoperative weight as the main predictor of postoperative body weight and preoperative size and liposuction-related factors as key predictors of postoperative circumferential size. The final model was integrated into a web-based CDSS (365mc AI platform).

**Conclusions:**

We developed and validated a chained multioutput regression model to predict postoperative body weight and circumferential size after liposuction. Integrated into a web-based CDSS, the model may support patient-specific preoperative counseling and surgical planning.

## Introduction

Liposuction is a widely performed cosmetic surgical procedure to remove localized fat deposits and enhance body contours [1]. The International Society of Aesthetic Plastic Surgery reported more than 5.1 million body contouring operations, including liposuction, in 2023, placing it among the 5 most frequent cosmetic surgeries worldwide [2]. As liposuction cases increase, patients increasingly seek individualized predictions of their postoperative results, whereas clinicians require comprehensive preoperative profiles to support data-driven surgical decision-making [3].

Despite these advances, postoperative outcomes after liposuction remain difficult to predict because they are influenced by patient-specific, anthropometric, and procedural factors. Recent machine learning (ML) studies have used patient-level data and ensemble methods to improve prediction of surgical or anthropometric outcomes [4-6]. However, evidence remains limited for liposuction-specific postoperative prediction using large multicenter datasets, and many studies have focused on single outcomes rather than modeling related targets together. As summarized in Table S1 in Multimedia Appendix 1, prior liposuction-related prediction studies were largely limited to single-center cohorts, single-outcome models, or algorithms that did not explicitly account for dependencies between correlated postoperative outcomes. To our knowledge, no previous study has jointly modeled postoperative body weight and circumferential size using a multicenter dataset of this scale.

Postoperative body weight and circumferential size are closely related outcomes after liposuction. Predicting them independently may overlook the relationship between overall weight change and local body-contour change. Previous multioutput learning studies have shown that when response variables are correlated or clinically dependent, chained regression models can be useful because they use the prediction of one target as additional information for another [7,8]. This structure allows the model to capture intertarget dependencies that may not be reflected in separate single-output models [9]. On the basis of this rationale, we developed a chained multioutput regression model to jointly predict postoperative body weight and circumferential size.

Therefore, this study aimed to develop and validate a chained multioutput ML framework to predict postoperative body weight and circumferential size in individuals undergoing liposuction. Using a large multicenter dataset from 20 obesity specialty hospitals in the 365mc network in South Korea, we evaluated a range of algorithms, including chained multioutput models, to jointly predict postoperative body weight and circumferential size while accounting for the relationship between these outcomes. The best-performing configuration was subsequently integrated into a web-based clinical decision support system (CDSS), enabling its use for preoperative planning and individualized patient counseling in clinical practice.

## Methods

### Patient and Data Collection

We analyzed anonymized, deidentified data from a multicenter cohort comprising individuals who underwent liposuction in 2024 across 20 obesity specialty clinics in the 365mc network in South Korea [10]. A comprehensive dataset was constructed from patients who underwent procedures at these clinics throughout 2024, totaling 8064 individuals. To ensure data integrity for model development, patients with incomplete records regarding the liposuction site or postoperative outcomes were excluded. After data preprocessing, the final cohort included 7804 patients. A detailed flowchart of the data processing is shown in Figure S1 in Multimedia Appendix 1.

### Ethical Considerations

The research protocol was approved by the institutional review board of Kyung Hee University (KHUH 2024–04–002). The requirement for informed consent was waived by the board, as deidentified data were used for all analyses. This study was conducted in accordance with the principles outlined in the Declaration of Helsinki. The Transparent Reporting of a Multivariable Prediction Model for Individual Prognosis or Diagnosis statement was followed to guide the design, analysis, and reporting of this study [11].

### Multicenter Liposuction Cohort

The multicenter liposuction cohort included individuals aged ≥17 years who underwent liposuction between 1 January and 31 December 2024 at 20 obesity specialty clinics in the 365mc network in South Korea. Clinical records from these branches were consolidated into a single dataset and randomly partitioned into training, validation, and test subsets for model development. The ML model was developed to simultaneously predict 2 primary postoperative outcomes: body weight and site-specific circumference, using preoperative variables [12]. Participants’ weight and the dimensions of the treated site were measured preoperatively and again at a mean of 3.82 (SD 2.24) weeks after liposuction with a bioelectrical-impedance analyzer; all measurements were obtained with the InBody 370S (InBody Co) [13]. Only patients with complete paired preoperative and postoperative anthropometric and bioelectrical data were included in the final analysis.

### Model Variables

To develop a multioutput ML-based model for predicting postoperative outcomes after liposuction, we used the following 15 features: sex (male or female), age (continuous), height (continuous), preoperative body weight (continuous), BMI (continuous), preoperative size (continuous), liposuction technique (local anesthetic minimally invasive liposuction [LAMS] or conventional surgical liposuction), liposuction site (abdomen, arms, back, buttocks, calves, flanks, or thighs), skeletal muscle mass (continuous), body fat mass (continuous), total body water (continuous), fat-free mass (continuous), body protein (continuous), body mineral content (continuous), and waist-to-hip ratio (continuous). Detailed descriptions of the variables are provided in Table S2 in Multimedia Appendix 1.

### Preprocessing

The dataset was randomly split into training and test sets in a 7:3 ratio. The test set was used only for final model evaluation and was not accessed during training or internal validation. Missing values were handled using multiple imputation by chained equations. Continuous variables were winsorized to reduce the influence of outliers [14], and z-score standardization was then performed using the mean and SD estimated from the training set, which were subsequently applied to the test set [15]. Feature selection was performed using least absolute shrinkage and selection operator (LASSO) regularization, with detailed procedures provided in Multimedia Appendix 1 and the selected features shown in Figure S2 in Multimedia Appendix 1. The correlation matrix of the resulting feature set is shown in Figure S3 in Multimedia Appendix 1 [16,17].

### ML Algorithms

We established a multioutput ML framework to predict postoperative body weight and circumferential size using preoperative profiles from the 365mc network. The candidate algorithms included tree-based ensemble models, including the random forest regressor and extra trees regressor; boosting-based models, including gradient boosting regressor (GBR), histogram-based GBR (HGBR), and adaptive boosting (AdaBoost) regressor; and a kernel-based model, support vector regressor [18,19]. The Tree-Based Pipeline Optimization Tool (TPOT; Epistasis Lab) was also evaluated as an automated ML approach [20]. Ensemble strategies, including the voting regressor and stacking regressor, were applied to combine predictions from multiple base learners [21]. For multioutput prediction, a regressor chain framework was implemented to model the dependency between postoperative body weight and circumferential size. The principles and key formulas of the algorithms used in this study are provided in Table S3 in Multimedia Appendix 1. To characterize the variability and distribution of the target variables, descriptive statistics were calculated separately for the training and test sets and are presented in Table S4 in Multimedia Appendix 1. All models were optimized using grid search [22], and the final hyperparameters are summarized in Table S5 in Multimedia Appendix 1.

### Multioutput Regression Model

Multioutput learning was used to jointly predict 2 related postoperative outcomes, postoperative body weight and circumferential size, from the same selected input features [23,24]; let x denote the predictor vector for a given patient, and let y=(y1, y2) denote the target outcomes. We implemented a wrapper-based regressor chain using the extra trees regressor as the base estimator, allowing the prediction of the first target to inform the second target [8]. In the forward chain, postoperative body weight was predicted first and then used as an additional feature to estimate circumferential size:


$$\left[{\hat{y}}_{1}=f_{1}(x)][{\hat{y}}_{2}=f_{2}(x,{\hat{y}}_{1})\right]$$

In the reverse chain, the order of the 2 targets was swapped. Both chain directions were evaluated to assess the influence of target ordering on model performance. The overall objective was to minimize the joint prediction error across both outcomes:


$$L=\frac{1}{N}\sum_{i=1}^{N}\left[\ell \left(y_{i1},{\hat{y}}_{i1}\right)+\ell \left(y_{i2},{\hat{y}}_{i2}\right)\right]$$

The extra trees regressor with the forward chain order was selected as the final multioutput prediction model based on its overall predictive performance.

### Model Training and Evaluation

Model performance was evaluated using 5-fold cross-validation on the training set, as described in Multimedia Appendix 1. Predictive performance was assessed using the coefficient of determination (*R*^2^), root mean square error (RMSE), mean absolute error (MAE), and mean absolute percentage error (MAPE), with their definitions and formulas summarized in Table 1 [25]. Higher *R*^2^ values and lower RMSE, MAE, and MAPE values indicated better performance. Model selection prioritized *R*^2^, with RMSE used as a secondary criterion among models with comparable *R*^2^ values; MAE and MAPE were additionally considered to provide a comprehensive assessment of predictive performance. Model interpretability was assessed using Shapley additive explanations (SHAP) values [26], and an ablation analysis was performed by sequentially removing the most influential predictors and evaluating the resulting changes in performance [27]. All analyses were conducted in Python (version 3.10.17; Python Software Foundation), NumPy (version 1.26.4; NumFOCUS), Pandas (version 1.5.3; NumFOCUS), Matplotlib (version 3.10.1; NumFOCUS), and Scikit-learn (version 1.6.1; NumFOCUS) [28].

**Table 1.** Four statistical metrics used for performance assessment of algorithm methods in this research.

Metrics	Description
*R*^2^=$1-\frac{\sum_{i=1}^{n}(Actual_{i}-Predicted_{i}{)}^{2}}{\sum_{i=1}^{n}(Actual_{i}-Actual_{avg}{)}^{2}}$	Coefficient of determination
MSE=$\frac{1}{n}\sum_{i=1}^{n}(Actual_{i}-Predicted_{i}{)}^{2}$	Mean squared error
RMSE=$\sqrt{\frac{1}{n}\sum_{i=1}^{n}(Actual_{i}-Predicted_{i}{)}^{2}}$	Root mean squared error
MAE=$\frac{1}{n}\sum_{i=1}^{n}\left|Actual_{i}-Predicted_{i}\right|$	Mean absolute error
MAPE=$\frac{100\%}{n}\sum_{i=1}^{n}\left|\frac{Actual_{i}-Predicted_{i}}{Actual_{i}}\right|$	Mean absolute percentage error

### Statistical Analysis

To evaluate the reliability of the model’s continuous predictions, agreement with the observed postoperative measurements was assessed using the Bland-Altman methodology [29]. For each patient, the difference between the predicted and observed values was plotted against their mean, and the overall mean difference together with the 95% limits of agreement (LoA) based on the SD was derived. Separate analyses were performed for postoperative body weight and circumferential size. To assess model robustness across different patient populations, subgroup analyses were performed according to sex and age group (Table S7 in Multimedia Appendix 1). To facilitate clinical adoption, an AI-driven, web-based CDSS was subsequently developed, enabling surgeons to input preoperative data and receive real-time predictions of postoperative body weight and circumferential size outcomes at the point of care [30].

## Results

### Study Population

The multicenter 365mc cohort comprised 8064 patients who underwent liposuction between January 1, 2024, and December 31, 2024, at 20 obesity specialty hospitals in the 365mc network in South Korea. After excluding 3.2% (260/8064) incomplete records, 96.8% (7804/8064) eligible participants were included in the final analysis, as shown in Figure S1 in Multimedia Appendix 1. The excluded records included 3.2% (249/7804) records with missing postoperative body weight or treated-site circumference, 0.1% (8/7804) records with missing surgical-site information, and 0.04% (3/7804) records with missing sex information. Baseline characteristics are summarized in Table 2. The cohort included 7612 (97.54%) female participants and 192 (2.46%) male participants. The mean age was 35.3 (SD 8.6) years, and the mean BMI was 24.0 (SD 3.9) kg/m^2^.

**Table 2.** Baseline characteristics of patients who underwent liposuction at a single branch within the 20-branch 365mc liposuction hospital network in South Korea, used to develop a multioutput machine learning model for predicting postoperative body weight and circumferential size (N=7804).

Variables	Participants
Sex, n (%)	
Male	192 (2.46)
Female	7612 (97.54)
Age (years), mean (SD)	35.25 (8.57)
Height (cm), mean (SD)	161.91 (5.35)
Preoperative weight (kg), mean (SD)	63.06 (11.38)
BMI (kg/m^2^), mean (SD)	23.99 (3.87)
Preoperative size (cm), mean (SD)	57.92 (25.82)
Liposuction technique, n (%)	
Local anesthetic minimally invasive liposuction	6832 (87.54)
Surgery	972 (12.46)
Liposuction site, n (%)	
Abdomen	1888 (24.19)
Arms	3455 (44.27)
Backs	332 (4.25)
Buttocks	126 (1.61)
Calves	96 (1.23)
Flanks	543 (6.96)
Thighs	1364 (17.48)
Skeletal muscle mass (kg), mean (SD)	22.33 (3.37)
Body fat mass (kg), mean (SD)	21.93 (7.69)
Total body water (kg), mean (SD)	30.15 (4.10)
Fat-free mass (kg), mean (SD)	41.14 (5.59)
Body protein (kg), mean (SD)	8.07 (1.12)
Body mineral (kg), mean (SD)	4.94 (7.47)
Waist-to-hip ratio, mean (SD)	0.89 (0.06)

### Model Performance

Table 3 summarizes the performance of all candidate ML models and approaches, evaluated using *R*^2^, RMSE, MAE, and MAPE. Using 15 predictors, we compared 8 individual ML models, an automated ML approach, 2 ensemble approaches, and chained multioutput regression models for predicting postoperative body weight and circumferential size. In the 5-fold cross-validation of the training dataset, the chained extra trees regressor with a forward prediction order showed the best overall performance across all evaluation metrics, with an *R*^2^ of 0.986 (95% CI 0.983-0.988), an RMSE of 1.916 (95% CI 1.680-2.151), an MAE of 1.042 (95% CI 1.006-1.078), and a MAPE of 1.839 (95% CI 1.765-1.913). The stacked model using the extra trees regressor and random forest regressor showed comparable performance, with an *R*^2^ of 0.985 (95% CI 0.983-0.988), an RMSE of 1.932 (95% CI 1.684-2.179), an MAE of 1.052 (95% CI 1.020-1.085), and a MAPE of 1.859 (95% CI 1.763-1.925).

**Table 3.** Predictive performance comparison of machine learning–based algorithms on the training and test datasets from the 365mc liposuction hospital network.

Algorithms	*R* ^2^	Root mean square error	Mean absolute error	Mean absolute percentage error
Training dataset (5-fold cross-validation), estimate (95% CI)				
Single				
Random forest regressor	0.983 (0.980-0.985)	2.080 (1.836-2.325)	1.188 (1.160-1.215)	2.097 (2.056-2.137)
Extra trees regressor	0.985 (0.983-0.986)	1.930 (1.686-2.174)	1.047 (1.015-1.078)	1.849 (1.785-1.913)
Gradient boosting regressor	0.982 (0.980-0.985)	2.072 (1.809-2.334)	1.284 (1.266-1.303)	2.281 (2.238-2.323)
Histogram-based gradient boosting regressor	0.983 (0.980-0.986)	2.040 (1.768-2.312)	1.236 (1.204-1.268)	2.190 (2.134-2.246)
AdaBoost^a^ regressor	0.963 (0.960-0.966)	3.083 (2.853-3.313)	2.151 (2.084-2.219)	3.918 (3.701‐4.136)
Support vector regressor	0.973 (0.969-0.978)	2.531 (2.234-2.829)	1.501 (1.455-1.546)	2.626 (2.537-2.714)
TPOT^b^: extra trees regressor and random forest regressor	0.985 (0.982-0.987)	1.961 (1.710-2.211)	1.102 (1.071-1.133)	1.948 (1.893-2.002)
Voting: extra trees regressor and random forest regressor	0.985 (0.982-0.987)	1.961 (1.710-2.211)	1.102 (1.071-1.133)	1.948 (1.893-2.002)
Stacked^c^: extra trees regressor and random forest regressor	0.985 (0.983-0.988)	1.931 (1.684-2.179)	1.062 (1.032-1.092)	1.877 (1.815-1.940)
Chained				
Extra trees regressor (forward order)^d^^,^^e^	*0.986* (*0.983-0.988*)	*1.916* (*1.680-2.151*)	*1.042* (*1.006-1.078*)	*1.839* (*1.765-1.913*)
Extra trees regressor (reverse order)^f^	0.985 (0.983-0.988)	1.932 (1.684-2.179)	1.052 (1.020-1.085)	1.859 (1.793-1.925)
Test dataset, estimate				
Single				
Random forest regressor	0.979	2.448	1.247	2.190
Extra trees regressor	0.980	2.390	1.117	1.962
Gradient boosting regressor	0.978	2.485	1.335	2.364
Histogram-based gradient boosting regressor	0.980	2.359	1.110	1.946
AdaBoost regressor	0.959	3.230	2.084	3.736
Support vector regressor	0.969	2.792	1.557	2.709
TPOT: extra trees regressor and random forest regressor	0.974	1.754	1.113	1.790
Voting: extra trees regressor and random forest regressor	0.980	2.380	1.168	2.052
Stacked^c^: extra trees regressor and random forest regressor	0.980	2.379	1.134	1.993
Chained				
Extra trees regressor (forward order)^d^^,^^e^	*0.980*	*2.356*	*1.242*	*2.199*
Extra trees regressor (reverse order)^f^	0.979	2.394	1.125	1.975

a AdaBoost: adaptive boosting.

b TPOT: Tree-Based Pipeline Optimization Tool; TPOT indicates a genetic programming–based automated machine learning framework.

c This stacking ensemble used extra trees regressor and random forest regressor as base learners, with ridge regressor as the meta-learner.

d The regressor chain using the extra trees regressor was implemented as a multioutput regression model, predicting postoperative body weight first, followed by size.

e Italicized data indicate the best performance.

f The regressor chain using the extra trees regressor was implemented as a multioutput regression model, predicting postoperative circumferential size first, followed by weight.

**Figure 1.**
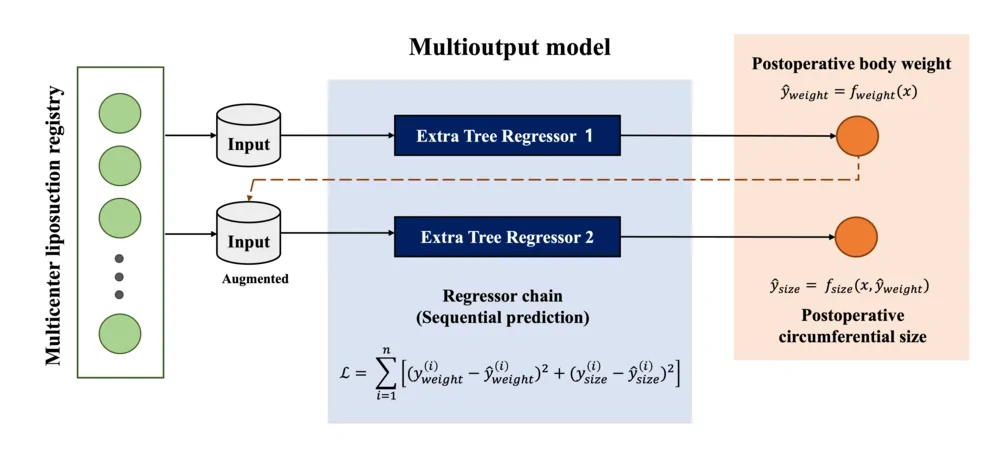
Architecture of the machine learning chain model for multioutput prediction of postoperative body weight and circumferential size.

**Figure 2.**
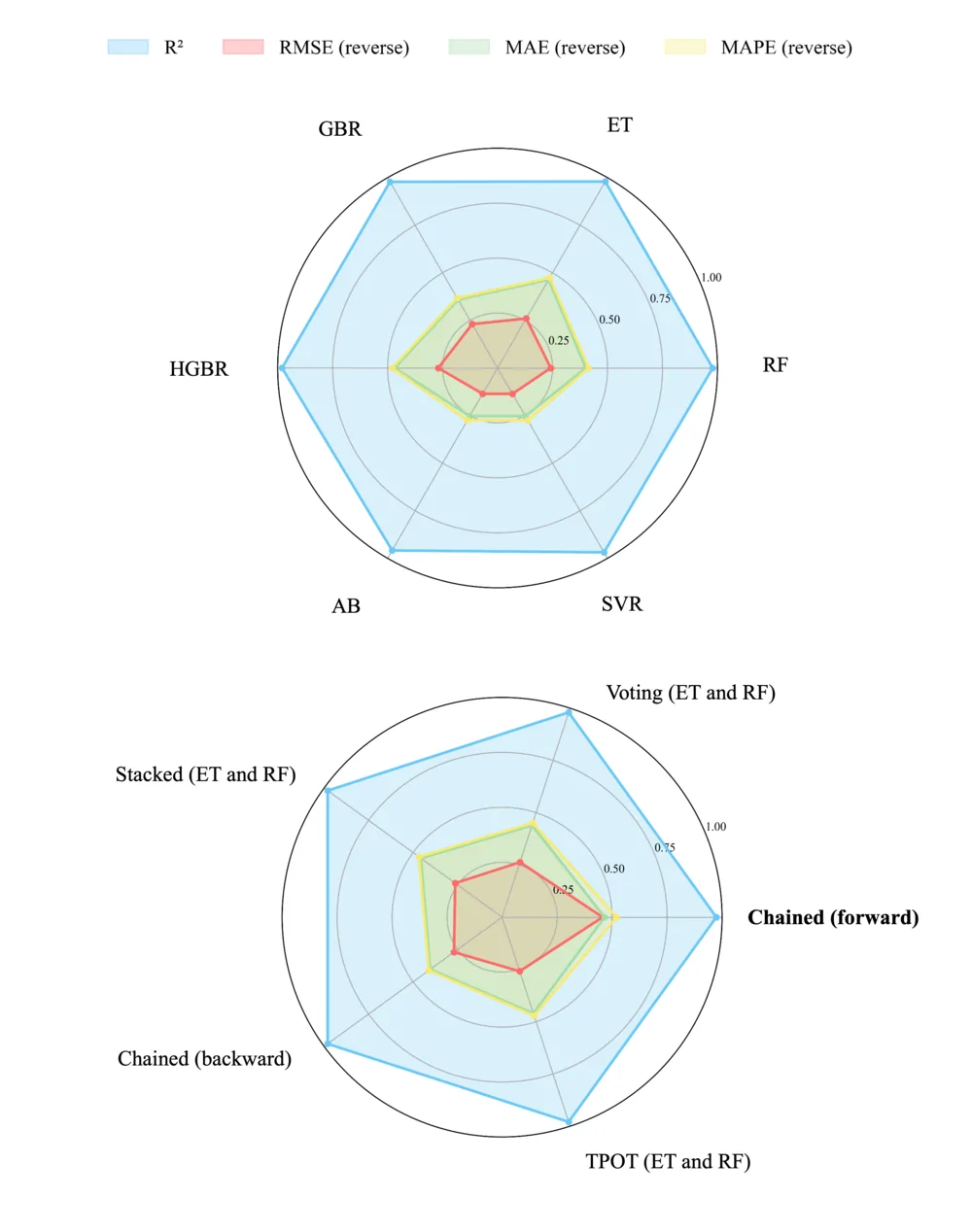
Performance comparison of all models for predicting postoperative outcomes. The root mean square error (RMSE), mean absolute error (MAE), and mean absolute percentage error (MAPE) values are shown as their inverse values in the radar charts. Bold text indicates the best-performing model, with values closer to 1 for all 4 metrics signifying better performance. AB: adaptive boosting; ET: extra trees; GBR: gradient boosting regressor; HGBR: histogram-based gradient boosting regressor; RF, random forest; SVR: support vector regressor; TPOT: Tree-Based Pipeline Optimization Tool.

**Figure 3.**
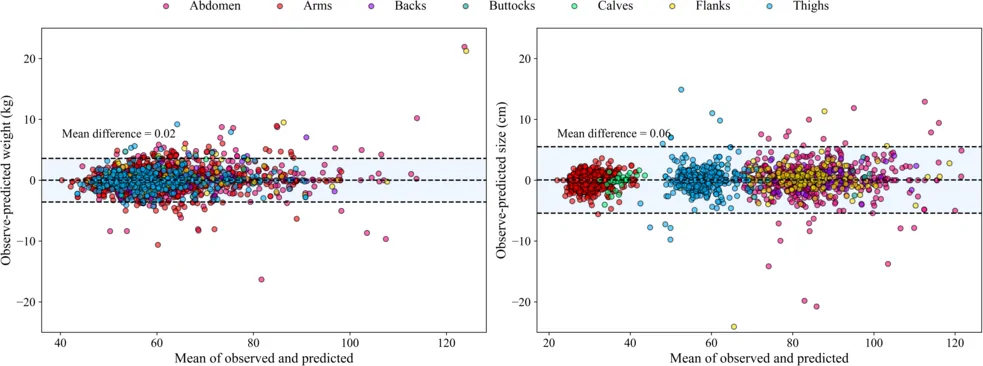
Bland-Altman plots for our proposed model between observed and predicted postoperative body weight and circumferential size across different liposuction sites. Each point represents a patient, color-coded by surgical site. The shaded regions indicate the mean difference and 95% limits of agreement (SD 1.96).

In the independent test dataset, the chained extra trees regressor with a forward prediction order achieved an *R*^2^ of 0.980, an RMSE of 2.356, an MAE of 1.242, and a MAPE of 2.199. Among the models achieving the highest *R*^2^ of 0.980, this model had the lowest RMSE and was therefore selected as the final model based on the model-selection criteria. HGBR showed comparable performance, with an *R*^2^ of 0.980, an RMSE of 2.359, and an MAE of 1.110. The architecture of the selected chained multioutput regression model is illustrated in Figures 1 and 2. To further evaluate predictive reliability, Bland-Altman analyses stratified by surgical site are presented in Figure 3.

### Feature Importance Based on SHAP Values

**Figure 4.**
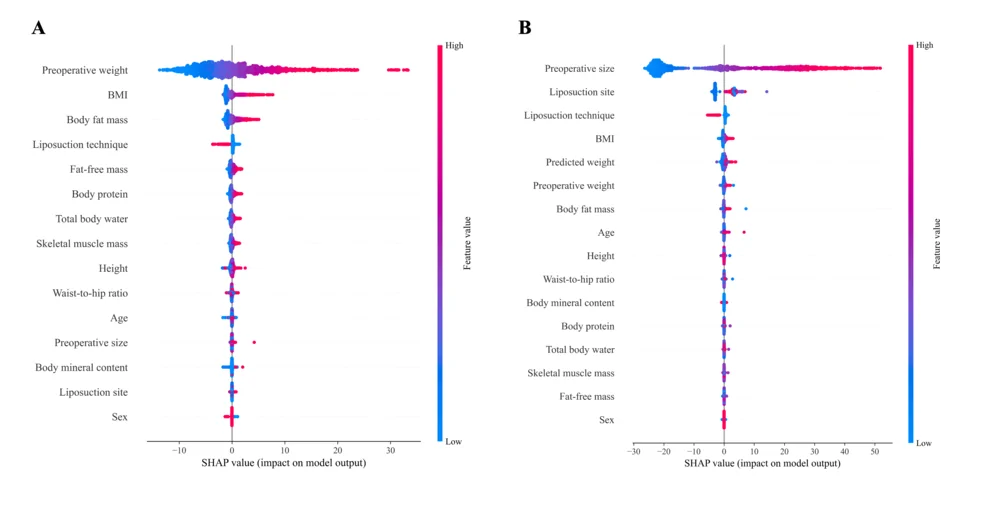
Shapley additive explanation (SHAP) values for the proposed regressor chain model for postoperative outcomes prediction. (A) First regressor predicting postoperative body weight and (B) second regressor predicting postoperative circumferential size, incorporating both original features and the predicted weight from the first regressor.

Figure 4 displays SHAP summary plots for the chained multioutput model. Figure 4A illustrates the first regressor, which predicts postoperative body weight, confirming that preoperative weight and related clinical variables exert the greatest influence on the output. Figure 4B depicts the second regressor, which incorporates the predicted postoperative body weight from the first stage as an additional feature when estimating postoperative circumferential size. In this second stage, the predicted weight becomes an important predictor alongside preoperative circumferential size, liposuction site, and surgical technique.

### Ablation Study

To quantify the marginal contribution of each key feature, we performed an ablation analysis by sequentially removing 4 influential features: preoperative size, liposuction site, liposuction technique, and BMI. Model performance was then reassessed after each feature removal. The exclusion of preoperative size resulted in the largest performance decrease, with *R*^2^ decreasing from 0.980 to 0.975 and error metrics increasing from 2.356 to 2.868 for RMSE, from 1.242 to 1.419 for MAE, and from 2.199% to 2.445% for MAPE. Despite this decrease, the model maintained strong predictive performance, suggesting that the overall prediction framework was robust to the removal of individual features. Full results of the ablation analysis are provided in Table S6 in Multimedia Appendix 1.

### Web-Based CDSS

**Figure 5.**
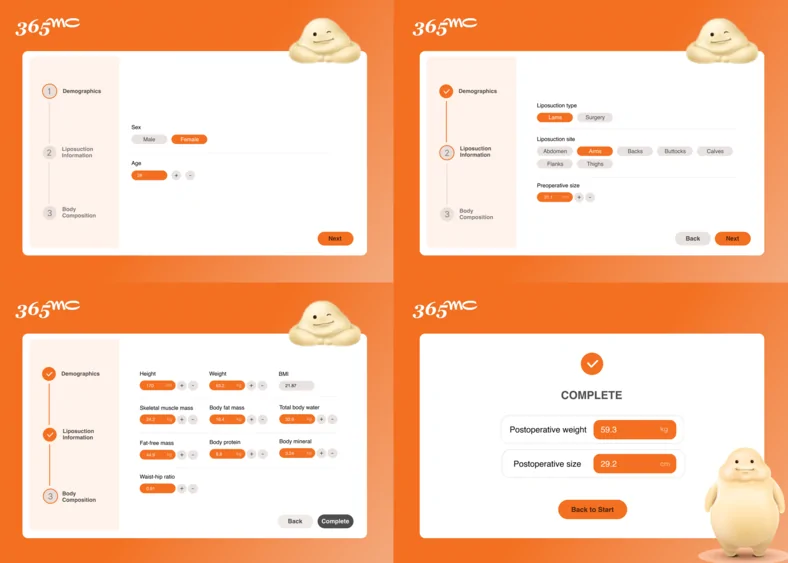
User interface of the web-based clinical decision support system for personalized postliposuction outcome prediction (365mc AI platform).

To facilitate clinical implementation, we deployed the chained multioutput model as a web-based CDSS accessible (365mc AI platform [31]). The platform guides the user through 3 sequential input screens that capture demographic details, operative variables, and body composition measurements, after which it instantly returns personalized forecasts of postoperative body weight and circumferential size. Figure 5 illustrates the interface, showing the stepwise data-entry process and the final results page. All information entered by the user is transmitted over an encrypted connection and is automatically deleted once the prediction is generated, ensuring that no personally identifying data are retained or stored on the server.

## Discussion

### Key Findings

This study developed and validated a chained multioutput ML model that jointly predicts postoperative body weight and circumferential size using 15 predictors from a multicenter liposuction cohort across 20 obesity specialty clinics in the 365mc network. Among the benchmarked single-output, automated ML, ensemble, and chained approaches, the chained extra trees regressor achieved the best performance (*R*^2^=0.980, RMSE=2.356), supporting the value of explicitly modeling the dependency between these two clinically related outcomes. In this structure, postoperative body weight was predicted first and then used to estimate postoperative circumferential size, underscoring the conditional relationship between the two outcomes. Feature-importance analysis showed that preoperative weight was the main predictor of postoperative body weight, whereas preoperative size, liposuction-related factors, and the predicted weight estimate most strongly influenced postoperative circumferential size. The model was implemented as a web-based CDSS, supporting its potential for preoperative planning and individualized patient counseling.

### Plausible Mechanism and Comparison With Previous Studies

This study proposed a chained multitarget regression model to jointly predict postoperative body weight and circumferential size after liposuction. These two outcomes are clinically related, and predicting them separately may fail to capture the dependency between overall weight change and local body-contour change [10,32]. Unlike previous single-target approaches, our model used the prediction of one outcome as additional information for predicting the other. This structure allowed the model to reflect the conditional relationship between postoperative body weight and circumferential size, which is important for surgical planning, objective outcome assessment, and patient counseling [33]. SHAP analysis further supported this relationship by showing that the predicted postoperative body weight contributed meaningfully to the prediction of postoperative circumferential size [34].

From a methodological perspective, direct single-output regression models and chained regression models have different strengths. Direct models are simple and useful when target outcomes are relatively independent, but they may not capture dependencies between clinically related outcomes [7]. In contrast, chained regression models use the prediction of one target as additional information for another, allowing them to reflect intertarget relationships [7,8]. In this study, postoperative body weight and circumferential size were considered clinically related outcomes after liposuction, and the chained extra trees regressor achieved the best overall performance, suggesting that modeling this dependency was appropriate for our prediction task. However, because predictions in subsequent steps depend on those made in the preceding steps, careful validation of the prediction order and model robustness is required [35].

Ensemble approaches also showed strong performance, with voting providing stable averaged predictions and stacking allowing a meta-model to combine base learners more flexibly [36,37]. However, more complex models, including stacking and chained regression, require careful validation because their performance may depend on the dataset structure, target relationships, and prediction order. Therefore, although our findings support the usefulness of the chained multioutput approach, future studies should further evaluate different chaining orders and validate the model in external datasets to confirm its generalizability and robustness.

### Clinical and Policy Implementation

Building on these methodological and ML modeling advances, we deployed our proposed chained multioutput model as a web-based CDSS that simultaneously forecasts postoperative body weight and circumferential size while leveraging individual patient profiles. By presenting key features including the surgical site, technique, and conditional relationship between outcomes, the system may enhance personalized preoperative counseling and surgical planning [38]. Explicit modeling and communication of the interdependence between outcomes further bolster interpretability and may increase clinician and patient trust, underscoring the need for prospective evaluation of how these explanatory elements affect decision quality, patient satisfaction, and adherence to recommended care [39].

### Limitations

Despite promising performance, this study has several limitations. First, the proposed model was developed using a retrospective cohort from a single high-volume liposuction network in South Korea, and the study population was predominantly young Asian women with a relatively low BMI [40]. These characteristics may restrict the external validity of the findings when applied to other ethnicities, anatomical surgical sites, or clinical settings. Second, reliance on existing medical records introduces potential selection bias and missing data; although records with critical omissions were excluded, residual confounding and incomplete capture of relevant variables cannot be excluded [41]. Third, although the model effectively leverages the conditional dependence between postoperative circumferential size and body weight, it does not explicitly characterize higher-order interactions among other preoperative factors such as age, sex, and body composition, which could further enhance predictive performance. Finally, the surgical-site information was based on a single, operator-designated representative region, and definitions or granularity of those regions may vary across institutions, limiting the generalizability of findings related to procedural localization. Prospective validation in multicenter and more diverse populations, including assessments across finer anatomical subregions and evaluation of the stability of key predictive features, is needed to confirm and extend these results.

### Conclusions

This study developed and validated a chained multioutput regression model to predict postoperative body weight and circumferential size using 15 predictors from a multicenter liposuction cohort across 20 obesity specialty clinics in the 365mc network. The model accounted for the dependency between the two postoperative outcomes and was implemented as a web-based CDSS. The key findings are as follows:

- The extra trees regressor–based chained multioutput regression model achieved an *R*^2^ of 0.980 and an RMSE of 2.356 and was selected as the final model based on its high *R*^2^ value and favorable RMSE value among models with comparable *R*^2^ values.
- Predicting postoperative body weight first and then using it to estimate circumferential size captured the conditional relationship between the two outcomes.
- Feature-importance analysis identified preoperative weight as the main predictor of postoperative body weight, while preoperative size and liposuction-related factors were most influential in predicting postoperative circumferential size.

## Supplementary material

10.2196/82145 Multimedia Appendix 1 L1 regularization and 5-fold cross-validation for the multi-output postoperative outcome prediction model, supplementary figures (study flowchart, L1 regularization feature selection, and correlation matrix), and supplementary tables (literature summary, feature descriptions, machine learning algorithms, distribution statistics, hyperparameters, ablation study, and subgroup analyses).

## Abbreviations

AdaBoost: adaptive boosting
CDSS: clinical decision support system
GBR: gradient boosting regressor
HGBR: histogram-based gradient boosting regressor
LAMS: local anesthetic minimally invasive liposuction
LASSO: least absolute shrinkage and selection operator
LoA: limits of agreement
MAE: mean absolute error
MAPE: mean absolute percentage error
ML: machine learning
RMSE: root mean square error
SHAP: Shapley additive explanation
TPOT: Tree-Based Pipeline Optimization Tool
