# Supplementary material for: Multioutput Machine Learning Model for Predicting Postoperative Outcomes After Liposuction: Algorithm Development and Validation Study in a Multicenter Cohort
Source: JMIR Med Inform. 2026 Sep 23;14:e82145. doi: 10.2196/82145 (PMC13600656; doi:10.2196/82145)
Supplement: Supplementary file 1 [file medinform-v14-e82145-s001.docx]

**Supplement Material**

# Original Article

**Multi-output machine learning model for predicting postoperative outcome after liposuction: an algorithm development and validation study in a multicenter cohort**

**Running title:** Post-liposuction outcome prediction

Chaewoo Lee,^1,2∥^ Seoyoung Park,^1,3∥^ Jiyoung Hwang,^1^ Selin Woo,^1^ Youn Chan Park,^4^ Jae Won Seo,^5^ Sun Ho Lee,^6^ Jae Hyun Ahn,^7^ Dong Keon Yon,^1,3,8*^ Sang Youl Rhee,^1,9*^

∥These authors contributed equally.

*Corresponding authors

**Sang Youl Rhee,** MD, PhD

Department of Endocrinology and Metabolism, Kyung Hee University College of Medicine, 23 Kyungheedae-ro, Dongdaemun-gu, Seoul 02447, South Korea

Email: bard95@hanmail.net

**Dong Keon Yon**, MD, PhD, FACAAI, FAAAAI, ATSF

Department of Pediatrics, Kyung Hee University College of Medicine, 23 Kyungheedae-ro, Dongdaemun-gu, Seoul, 02447, South Korea

Email: yonkkang@gmail.com

**Table of Contents**

[**Figure S1**. Flowchart for study selection.	3](#_Toc236266315)

[**Figure S2**. Number of selected features across different L1 regularization for predicting postoperative size and weight.	4](#_Toc236266316)

[**Figure S3**. Correlation matrix of the input features used to predict postoperative body size and weight, based on a multicenter cohort from 365MC liposuction hospitals.	5](#_Toc236266317)

[**Table S1**. Summary of previous algorithm study	6](#_Toc236266318)

[**Table S2**. Description of features used in the multicenter cohort from 365MC liposuction hospitals.	8](#_Toc236266319)

[**Table S3**. Summary of machine-learning algorithms used in this study	9](#_Toc236266320)

[**Table S4**. Statistical distributions of postoperative weight and circumference.	11](#_Toc236266321)

[**Table S5**. Optimized hyperparameters for six machine learning models.	12](#_Toc236266322)

[**Table S6**. Ablation study comparing model performance when excluding individual features.	13](#_Toc236266323)

[**Table S7**. Subgroup analysis of model performance for postoperative weight and circumference according to sex and age group.	14](#_Toc236266324)

[**Supplementary Methods** L1 regularization	15](#_Toc236266325)

[**Supplementary Methods** k-fold cross validation	16](#_Toc236266326)

[**Reference**	18](#_Toc236266327)

Figure S1. Flowchart for study selection. * The primary postoperative outcomes were defined as body weight and treated-site circumference measured after liposuction.

Figure S2. Number of selected features across different L1 regularization for predicting postoperative size and weight. The optimal α value determined via 5-fold cross-validation was 0.001 for both targets.

Figure S3. Correlation matrix of the input features used to predict postoperative body size and weight, based on a multicenter cohort from 365MC liposuction hospitals. BFM, body fat mass; BMI, body mass index; FFM, fat-free mass; SMM, skeletal muscle mass; TBW, total body water; WHR, waist-hip ratio.

Table S1. Summary of previous algorithm study

| **Independent variables** | **Target variable** | **Model** | **Metrics** | **Reference** |
| --- | --- | --- | --- | --- |
| Demographic data, Preoperative JOA score, Ambulatory status, Preoperative neurological deficits, Duration of symptoms, Operated level, No. of decompression levels, Bleeding loss, Poor quality of preoperative SSEP, Poor quality of intraoperative SSEP/MEP baseline, Comorbidities | Postoperative JOA Score | LightGBM, XGBoost, Random Forest, Naïve Bayes, Elastic Net | R², RMSE, MAE | Liu, Tun, et al.^1^ |
| Demographic data, Type of surgery, Histologic features, Tumor stage, Cancer location, Blood test results, Comorbidities, COPD medication use, Treatment modalities, Pre-bronchodilator PFT results | postoperative FEV1 | OLS, LASSO, Ridge, ElasticNet, Random Forest, XGBoost, LightGBM | R², MSE | Kwon, Oh Beom, et al.^2^ |
| Demographic data, Duration of diabetes and hypertension, Blood pressure, Laboratory values, Comorbidities, Drugs prescribed, Complications associated with DKD | eGFR | Linear Regression, Random Forest Regressor, Gradient Boost Regressor | R², Adjusted R², MAE, MSE, RMSE | Nayak, Sandhya, et al.^3^ |
| Landsat spectral bands, NDVI, EVI, NDWI, BSI, NDTI, NIRv, FAPAR, MODIS Land Surface Temperature, MODIS Water Vapor, Elevation, Slope, Hillshade, Positive Openness, Negative Openness, Geomorphons, Geometric temperature transformations | Median vegetation height | Ensemble Gradient Boosted Tree, LightGBM | R², RMSE, D^2^, CCC | Hunter, Maria O., et al.^4,5^ |
| Water-Cement ratio, Fly ash, Coarse aggregate, Fine aggregate, Super-plasticizer, Fiber diameter, Fiber length, Fiber volume fraction | Compressive strength, Flexural strength, Splitting tensile strength | XGBoost, Gradient Boosting, Random Forest, LightGBM, AdaBoost, KNN | R², MAE, MSE, RMSE, MAPE | Najmoddin, Alireza, Hossein Etemadfard, and Mansour Ghalehnovi.^6^ |
| Genotype, Planting date | Branch number, Growth period, Boll number, Seed number per plant | Random forest, MLP | R², RMSE, MAPE, MBE | Fakhrzad, Fazilat, et al.^7^ |
| Inlet air temperature, Inlet air relative humidity, Dry side air flow, Wet-to-dry side air flow ratio | Supply temperature, Total cooling capacity, Useful cooling capacity, Web-bulb effectiveness | Polynimial Regression | R², RMSE | Hua, Bian, et al.^7^ |
| Pressure, Temperature, Choke opening levels | Oil flow rate, Water flow rate, Gas flow rate | Support Vector Machine, Gaussian Process | MAPE | Wahid, Md Ferdous, et al^8^ |

**Abbreviation:** BSI, bare soil index; CCC, concordance correlation coefficient; COPD, chronic obstructive pulmonary disease; D², D² regression score; DKD, diabetic kidney disease; eGFR, estimated glomerular filtration rate; EVI, enhanced vegetation index; FAPAR, fraction of absorbed photosynthetically active radiation; FEV₁, forced expiratory volume in 1 second; JOA, Japanese Orthopedic Association; KNN, K-nearest neighbors; LASSO, least absolute shrinkage and selection operator; LightGBM, light gradient boosting machine; MEP, motor-evoked potentials; MLP, multilayer perceptron; MODIS, moderate resolution imaging spectroradiometer; NDTI, normalized difference tillage index; NDVI, normalized difference vegetation index; NDWI, normalized difference water index; NIRv, near-infrared reflectance of vegetation; OLS, ordinary least squares; PFT, pulmonary function test; Ridge, Ridge regression; SSEP, somatosensory-evoked potentials; XGBoost, extreme gradient boosting.

Table S2. Description of features used in the multicenter cohort from 365MC liposuction hospitals.

| **Variables** | **Type** | **Description** |
| --- | --- | --- |
| Sex | 2 categories | - Male |
|  |  | - Female |
| Age | Numerical | years |
| Height | Numerical | cm |
| Preoperative weight | Numerical | kg |
| BMI | Numerical | ${kg/m}^{2}$ |
| Preoperative size | Numerical | cm |
| Liposuction technique | 2 categories | - LAMS |
|  |  | - Surgery |
| Liposuction site | 7 categories | - Abdomen |
|  |  | - Arms |
|  |  | - Backs |
|  |  | - Buttocks |
|  |  | - Calves |
|  |  | - Flanks |
|  |  | - Thighs |
| Skeletal muscle mass | Numerical | kg |
| Body fat mass | Numerical | kg |
| Total body water | Numerical | kg |
| Fat-free mass | Numerical | kg |
| Body protein | Numerical | kg |
| Body mineral | Numerical | kg |
| Waist-hip ratio | Numerical | ratio |

Abbreviation: BMI, body mass index; LAMS, local anesthetic minimal-invasive liposuction.

Table S3. Summary of machine-learning algorithms used in this study

| **Type** | **Algorithm** | **Principle** | **Key formula** | | **Reference** |
| --- | --- | --- | --- | --- | --- |
| Single | Random Forest Regressor | Bootstrap aggregation of multiple decision trees with random feature selection; predictions are averaged across trees to reduce variance and improve generalizability. | ŷ = (1/T) Σᵗ fₜ(x) | | Revathy, G., et al.^9^ |
| Single | Extra Trees Regressor | Ensemble of randomized decision trees that uses random split thresholds; reduces variance through averaging while increasing tree diversity. | ŷ = (1/T) Σᵗ fₜ(x) | | Ahmad, Muhammad Waseem, Jonathan Reynolds, and Yacine Rezgui^10^ |
| Single | Gradient Boosting Regressor | Sequentially adds weak learners to correct residual errors from previous learners using additive optimization. | Fₘ(x) = Fₘ₋₁(x) + ηhₘ(x) | | Zhang, Zhendong, and Cheolkon Jung^11^ |
| Single | Histogram-based Gradient Boosting Regressor | Gradient boosting implementation that discretizes continuous features into histogram bins to reduce computational cost while maintaining boosting-based additive updates. | Fₘ(x) = Fₘ₋₁(x) + ηhₘ(x) | | Kazemi, Farzin, Neda Asgarkhani, and Robert Jankowski.^12^ |
| Single | Adaboost Regressor | Adaptive boosting that sequentially updates learner weights so that observations with larger prediction errors receive greater influence in subsequent learners. | F(x) = Σᵐ αₘhₘ(x) | | Ying, Cao, et al.^13^ |
| Single | Support Vector Regressor | Kernel-based regression that estimates a function within an epsilon-insensitive margin while controlling model complexity. | min ½\|\|w\|\|² + CΣᵢ(ξᵢ + ξᵢ*) | | Balcıoğlu, H. Ersen, and Ahmet Çağdaş Seçkin.^14^ |
| AutoML | TPOT | Genetic programming-based AutoML framework that optimizes preprocessing steps, model selection, and hyperparameters according to cross-validation performance. | P* = arg max₍P∈𝒫₎ CV(P) | | Shafighfard, Torkan, et al.^15^ |
| Ensemble | Voting Regressor | Combines multiple base regressors by averaging their predictions, thereby stabilizing predictions across model families. | ŷ = (1/M) Σᵐ ŷₘ | | Ghosh, Prantik, Abhivesh Shukla, and Fitri Yakub.^16^ |
| Ensemble | Staking Regressor | Uses predictions from multiple base learners as inputs to a meta-learner, which learns how to combine base-model predictions. | ŷ = g(ŷ₁, ŷ₂, …, ŷM) | | Li, Guoqing, et al.^17^ |
| Multi-output | Regressor Chain | Predicts multiple outcomes sequentially; previously predicted outcomes are used as additional inputs for subsequent target predictions. | | ŷⱼ = fⱼ(x, ŷ₁, …, ŷⱼ₋₁) | Wu, Zipeng, and Guan Lian.^18^ |

Abbreviations: AutoML, automated machine learning; CV, cross-validation; TPOT, Tree-based Pipeline Optimization Tool.

Table S4. Statistical distributions of postoperative weight and circumference.

| **Dataset** | **Target variable** | **N** | **Mean ± SD** | **Median [IQR]** | **Min–Max** | **Skewness** | **Kurtosis** | **IQR outliers, n (%)** |
| --- | --- | --- | --- | --- | --- | --- | --- | --- |
| Training set | Postoperative weight, kg | 5462 | 61.33 ± 11.37 | 58.80 [53.60–66.10] | 39.60–136.00 | 1.56 | 3.61 | 260 (4.76%) |
| Training set | Postoperative circumference, cm | 5462 | 55.00 ± 25.56 | 54.00 [29.80–79.50] | 22.00–134.50 | 0.35 | -1.3 | 0 (0.00%) |
| Test set | Postoperative weight, kg | 2342 | 61.29 ± 10.87 | 59.00 [53.80–65.80] | 40.30–134.70 | 1.51 | 3.71 | 107 (4.57%) |
| Test set | Postoperative circumference, cm | 2342 | 54.82 ± 25.43 | 54.50 [29.73–79.00] | 22.00–124.00 | 0.37 | -1.26 | 0 (0.00%) |

Abbreviations: IQR, interquartile range; SD, standard deviation.
The values are presented as mean ± standard deviation or median, as appropriate. Postoperative circumference measurements were taken at multiple sites (abdomen, arms, thighs, hips, and calves), which explains the wide variation in values. Outliers were defined using the 1.5 × interquartile range rule.

Table S5. Optimized hyperparameters for six machine learning models.

| **Model** | **Hyperparameters** |
| --- | --- |
| Random forest regressor | Minimum samples per leaf 1, minimum samples per split 2, and number of tree estimation with 100, and the maximum depth is set to *None.* |
| Extra trees regressor | Minimum samples per leaf 1, minimum samples per split 2, and number of tree estimation with 100, and the maximum depth is set to *None.* |
| Gradient boosting regressor | Booster type of 200 gradient boosted regression tree with maximum depth 5, learning rate with 0.1, minimum samples per split 2, and minimum samples per leaf 2. |
| Histogram-based gradient boosting regressor | Booster type of 200 gradient boosted regression tree with maximum depth 5, learning rate with 0.1, minimum samples per split 63, and minimum samples per leaf 20. |
| AdaBoost regressor | 200 decision-tree weak learners (each with maximum depth 3) trained by stage-wise boosting with a learning rate 0.01. |
| Support vector regressor | Radial-basis-function kernel with C = 10, ε = 0.1, and γ set to “scale” (1 / [number of features × variance]). |

Abbreviation: AdaBoost, adaptive boosting.

Table S6. Ablation study comparing model performance when excluding individual features.

| **Feature** | **R²** | **RMSE** | **MAE** | **MAPE** |
| --- | --- | --- | --- | --- |
| Full model | 0.980 | 2.356 | 1.242 | 2.199 |
| Preoperative size | 0.975 | 2.868 | 1.419 | 2.445 |
| Liposuction site | 0.496 | 13.459 | 9.836 | 22.047 |
| Liposuction technique | 0.495 | 13.500 | 9.845 | 22.086 |
| Body mass index | 0.496 | 13.482 | 9.851 | 22.130 |

Abbreviation: MAE, mean absolute error; MAPE, mean absolute percentage error; R^2^, R-squared; RMSE, root mean square error.

Table S7. Subgroup analysis of model performance for postoperative weight and circumference according to sex and age group.

| **Subgroup** | **N** | **Weight  R²** | **Weight  RMSE** | **Weight  MAE** | **Circumference  R²** | **Circumference RMSE** | **Circumference MAE** |
| --- | --- | --- | --- | --- | --- | --- | --- |
| Sex |  |  |  |  |  |  |  |
| Male | 61 | 0.919 | 4.457 | 2.098 | 0.907 | 4.188 | 2.515 |
| Female | 2,281 | 0.971 | 1.704 | 1.033 | 0.988 | 2.743 | 1.123 |
| Age |  |  |  |  |  |  |  |
| <30 years | 628 | 0.971 | 1.814 | 1.108 | 0.977 | 3.474 | 1.169 |
| 30–39 years | 1,100 | 0.977 | 1.650 | 1.014 | 0.993 | 2.142 | 1.056 |
| 40–49 years | 407 | 0.973 | 1.712 | 0.970 | 0.982 | 3.437 | 1.367 |
| ≥50 years | 207 | 0.945 | 2.767 | 1.345 | 0.994 | 1.967 | 1.273 |

Abbreviation: MAE, mean absolute error; R^2^, R-squared; RMSE, root mean square error.

Supplementary Methods L1 regularization

Least absolute shrinkage and selection operator (LASSO), also known as L1 regularization, is a regularization technique that adds a penalty term proportional to the L1 norm of the model coefficients.^19^ This penalty constrains the magnitude of the coefficients and helps reduce model overfitting by discouraging overly complex models. Because L1 regularization can shrink some coefficients exactly to zero, it also performs implicit feature selection by excluding less informative predictors from the model.^20^ In this study, L1 regularization was applied to reduce model complexity and improve generalizability by penalizing large coefficients and selecting the most relevant features. The objective function for L1-regularized regression can be expressed as follows:

$${Loss}_{L1}=MSE+ \lambda\sum_{i=1}^{n} \left| w_{i} \right|$$

Where:

- MSE = the mean squared error
- $w_{i}$ = the model coefficient for $i^{th}$ feature.
- $\lambda$ = the regularization parameter that controls the strength of the penalty
- $n$ = the number of features

Supplementary Methods k-fold cross validation

K-fold cross-validation is a statistical resampling technique used to evaluate the predictive performance and generalizability of a machine-learning model.^21^ In this approach, the entire dataset is divided into (K) approximately equal-sized subsets, referred to as folds. During each iteration, the model is trained using (K-1) folds and validated on the remaining fold. This process is repeated (K) times, with each fold used once as the validation set. The final model performance is calculated by averaging the performance metrics obtained across all (K) iterations.^22^

In each iteration, four folds are used for model training, and the remaining fold is used for validation. The validation fold is rotated across iterations, and the final performance is estimated as the average performance across all five validation results. In this study, 5-fold cross-validation was applied to assess model performance. Specifically, the dataset was divided into five folds.

This approach provides several advantages. First, it allows the model to be evaluated on multiple validation subsets, thereby reducing dependence on a single train-validation split. Second, it provides a more stable and reliable estimate of predictive performance by averaging results across repeated iterations. Third, it enables efficient use of the available dataset, which is particularly useful when the sample size is limited. Overall, 5-fold cross-validation was used to reduce potential bias in model evaluation and to better assess the generalizability of the developed models.^23^

Reference

1	Liu, T. *et al.* Development and Internal Validation of Machine Learning to Predict Postoperative Worse Functional Status after Surgical Treatment for Thoracic Spinal Stenosis. *Medical Science Monitor: International Medical Journal of Experimental and Clinical Research* **30**, e945310 (2024).

2	Kwon, O. B. *et al.* Prediction of postoperative lung function in lung cancer patients using machine learning models. *Tuberculosis and Respiratory Diseases* **86**, 203 (2023).

3	Nayak, S. *et al.* Development of a machine learning-based model for the prediction and progression of diabetic kidney disease: A single centred retrospective study. *International Journal of Medical Informatics* **190**, 105546 (2024).

4	Hunter, M. O. *et al.* Global 30-m annual median vegetation height maps (2000–2022) based on ICESat-2 data and Machine Learning. *Scientific Data* **12**, 1470 (2025).

5	Hua, B. *et al.* Developing an experiment-based strong machine learning model for performance prediction and full analysis of Maisotsenko dewpoint evaporative air cooler. *Energy* **310**, 133179 (2024).

6	Najmoddin, A., Etemadfard, H. & Ghalehnovi, M. Multi-output machine learning for predicting the mechanical properties of BFRC. *Case Studies in Construction Materials* **20**, e02818 (2024).

7	Fakhrzad, F., ShariffAl-Sheikh, W. M., Mohammed, M. M. & Meftahizadeh, H. Machine learning models for predicting morphological traits and optimizing genotype and planting date in roselle (Hibiscus Sabdariffa L.). *Scientific Reports* **15**, 29148 (2025).

8	Wahid, M. F., Tafreshi, R., Khan, Z. & Retnanto, A. Multiphase flow rate prediction using chained multi-output regression models. *Geoenergy Science and Engineering* **231**, 212403 (2023).

9	Revathy, G. *et al.* Random Forest Regressor based superconductivity materials investigation for critical temperature prediction. *Materials Today: Proceedings* **66**, 648-652 (2022).

10	Ahmad, M. W., Reynolds, J. & Rezgui, Y. Predictive modelling for solar thermal energy systems: A comparison of support vector regression, random forest, extra trees and regression trees. *Journal of cleaner production* **203**, 810-821 (2018).

11	Zhang, Z. & Jung, C. GBDT-MO: Gradient-boosted decision trees for multiple outputs. *IEEE transactions on neural networks and learning systems* **32**, 3156-3167 (2020).

12	Kazemi, F., Asgarkhani, N. & Jankowski, R. Machine learning-based seismic response and performance assessment of reinforced concrete buildings. *Archives of Civil and Mechanical Engineering* **23**, 94 (2023).

13	Ying, C., Qi-Guang, M., Jia-Chen, L. & Lin, G. Advance and prospects of AdaBoost algorithm. *Acta Automatica Sinica* **39**, 745-758 (2013).

14	Balcıoğlu, H. E. & Seçkin, A. Ç. Comparison of machine learning methods and finite element analysis on the fracture behavior of polymer composites. *Archive of Applied Mechanics* **91**, 223-239 (2021).

15	Shafighfard, T., Bagherzadeh, F., Rizi, R. A. & Yoo, D.-Y. Data-driven compressive strength prediction of steel fiber reinforced concrete (SFRC) subjected to elevated temperatures using stacked machine learning algorithms. *Journal of Materials Research and Technology* **21**, 3777-3794 (2022).

16	Ghosh, P., Shukla, A. & Yakub, F. in *2024 second international conference on advances in information technology (ICAIT).* 1-6 (IEEE).

17	Li, G. *et al.* Grid search based multi-population particle swarm optimization algorithm for multimodal multi-objective optimization. *Swarm and Evolutionary Computation* **62**, 100843 (2021).

18	Wu, Z. & Lian, G. in *2020 International Joint Conference on Neural Networks (IJCNN).* 1-10 (IEEE).

19	Ng, A. Y. in *Proceedings of the twenty-first international conference on Machine learning.* 78.

20	Tu, K.-C. *et al.* Predicting emergency mortality risk in traumatic brain injury: comparative analysis of machine learning and large language model GPT-5. *International Journal of Medical Informatics*, 106268 (2026).

21	Rodriguez, J. D., Perez, A. & Lozano, J. A. Sensitivity analysis of k-fold cross validation in prediction error estimation. *IEEE transactions on pattern analysis and machine intelligence* **32**, 569-575 (2009).

22	Gershov, S. *et al.* Detection of Microbehavior Intervals for Predicting Mental Health: Clinically Relevant and Advanced Multimodal Temporal Analysis. *Journal of Medical Internet Research* **28**, e87049 (2026).

23	Wong, T.-T. & Yeh, P.-Y. Reliable accuracy estimates from k-fold cross validation. *IEEE Transactions on Knowledge and Data Engineering* **32**, 1586-1594 (2019).
